# Organizational Challenges in the Pediatric Onco-hematology Units During the First and Second Wave of the COVID-19 Pandemic: A National Survey in Italy

**DOI:** 10.1007/s44228-022-00010-w

**Published:** 2022-06-23

**Authors:** Matteo Amicucci, Valentina Biagioli, Elena Rostagno, Marta Canesi, Anna Bergadano, Debora Botta, Moreno Crotti Partel

**Affiliations:** 1 Department of Onco Haematology and Cell and Gene Therapy, Bambino Gesù Children’s Hospital, IRCCS, Piazza Sant’Onofrio Square, 4, 00165 Rome, Italy; 2 Department of Biomedicine and Prevention, University of Rome “Tor Vergata”, Via Montpellier, 1, Rome, Italy; 3 Professional Development, Continuing Education and Research Service, Bambino Gesù Children’s Hospital, IRCCS, Piazza S. Onofrio, 4, 00165 Rome, Italy; 4 Pediatric Oncology and Hematology Unit, IRCCS Azienda Ospedaliero Universitaria di Bologna, Via Massarenti, 11, 40138 Bologna, Italy; 5 Pediatric Hematology and BMT Unit, Fondazione MBBM Onlus - Clinica Pediatrica Università degli Studi Milano Bicocca, Via Pergolesi, 33, 20900 Monza, Italy; 6 Pediatric Hematology and Oncology Stem Cell Transplantation and Cellular Therapy Division, Presidio Infantile Regina Margherita, Piazza Polonia, 94, 10126 Turin, Italy; 7 Pediatric Unit, Ospedale Santissima Annunziata-Asl Cn1, Via degli Ospedali 14, 12038 Savigliano, Italy; 8 Bachelor of Science in Nursing, ASST Spedali Civili - University of Brescia, Viale Europa, 15, 25123 Brescia, Italy

**Keywords:** Cancer, COVID-19, Pediatrics, Oncology, Hematology

## Abstract

This study aimed to describe and compare, at a national level, the measures implemented in the pediatric onco-hematology units and the number of infections among patients and healthcare staff during the first and second wave of the COVID-19 pandemic in Italy. A multicenter, descriptive, online survey was conducted between15th March and 15th April 2020 (T1) and between 1 and 31st January 2021 (T2). All the Italian Pediatric Oncology and Hematology Association (AIEOP) centers were invited to participate in the study. Data of the pre-pandemic, first, and second phase were compared. Thirty-six of the 48 AIEOP centers completed the survey (75%). Several organizational, screening, and swab measures were implemented by AIEOP centers to prevent the SARS-CoV-2 infection among patients and visitors. During the pandemic, there was a significant reduction in the number of onco-hematology inpatient beds (p < 0.001), including inpatient beds dedicated to hematopoietic stem cell transplantation (HSCT), and consultations in the outpatient clinics (p < 0.001). During the first wave, 37 pediatric patients with cancer tested positive for SARS-CoV-2 versus 174 patients during the second wave. The reduction in routine services was also greater in the second than in the first wave. All the AIEOP centers showed the capacity to adapt and promptly respond to both waves of the pandemic.

## Introduction

The consequences of the current SARS-CoV-2 pandemic on health care, infrastructure and socio-economic life have been significant and are likely to be long-lasting. The high contagiousness of this new viral agent, the high frequency of asymptomatic carriers, the lack of herd immunity, and a series of rare and severe clinical manifestations represent major challenges that are putting a strain on health systems worldwide. The disease, especially in its most severe clinical form, primarily affects older age adults, while healthy children and young adults are relatively spared. The national update on July 14, 2021 reported 4,257,667 cases and 127,028 deaths from COVID-19 in Italy. The pediatric population comprised 234,188 cases in the 0–9 age group (5.5% of the total), of which 12 died, and 413,151 cases in the 10–19 age group (9.7% of the total), of which 16 died [1]. The clinical course of SARS-CoV-2 infection in children with cancer and who are immunosuppressed does not appear to be more severe compared to healthy children of the same age [2, 3].

During the SARS-CoV-2 pandemic, a rapid and complex reorganization of departments and services was necessary, both to prevent the spread of the infection and to ensure timely access to diagnostic and therapeutic paths for new patients [4], while continuing treatment for current patients [5]. Within the Italian Association of Pediatric Oncology and Hematology (AIEOP) network (), a series of preventive measures have been implemented in order to contain the spread of the infection, including restrictions of visitors’ entry to clinical units, and reduction of educational and recreational activities dedicated to patients. Families and parent associations became more aware of protective isolation measures for home and hospital patients, as well as for residential facilities. Strict hygiene measures have been implemented with particular attention to hand washing and the implementation of Personal Protective Equipment (PPE) wearing by health care professionals, patients and caregivers when needed. Dedicated pathways have been identified for patients with SARS-CoV-2 or suspected disease, and preventive screening by performing nasal swabs. Non-urgent or deferrable examinations and visits have been reorganized, and health care teams or new departments specifically dedicated to COVID-19 positive patients have been created [5, 6].

During the initial waves of the pandemic in 2020 and 2021, with an increase in the number of patients found positive, it became necessary to find strategies, no longer for an emergency context, but for a longer and unspecified period to ensure high standards of care. Here, we present the data collected through a national survey conducted in the Italian pediatric onco-hematology centers belonging to the AIEOP network, in order to describe and compare the measures implemented, and the number of infections among patients and healthcare staff during the first and second wave of the COVID-19 pandemic.

## Methods

### Study Design, Sample and Setting

This is a descriptive, longitudinal multicenter study involving pediatric onco-hematology centers that are part of the AIEOP network. The study was carried out by the AIEOP Nursing Group. A formal email was sent to each center’s director and to the nursing contact (if any), inviting them to fill in an online survey. Data were collected at two time points: after the World Health Organization (WHO) declaration of a global pandemic between March 15th- April 15th, 2020 (T1) and during the second wave of the pandemic in Italy between January 1st-31st, 2021 (T2). Information about the pre-pandemic phase (T0) were also collected at the first time point.

### Instruments

The survey was devised by an expert panel of AIEOP nurses, taking into account the literature on isolation precautions [7] and the main directives on oncology care during the COVID-19 pandemic from the Italian Ministry of Health (IT-MoH) [8]. The content validity of each item of the survey was evaluated by 10 members of the AIEOP Nurses Group. The provision of informed consent to participate in the study was necessary to fill in the questionnaire. The survey consisted of 50 items divided into four main sections: (1) information about the center and its inpatient and outpatient workload; (2) isolation practices and protective measures adopted to prevent the COVID-19 outbreak; (3) testing facilities for patients and healthcare providers, PPE availability and utilization, staff management; (4) general considerations of critical or valuable experiences. In addition, responding centers were asked to report the number of COVID-19 positive cases among healthcare providers and patients.

### Data Analysis

The principal investigators verified missing or inconsistent data by contacting the responding center via telephone or e-mail. All data were managed in an aggregate way according to the privacy regulation. Data were analyzed from centers who completed at least 80% of the survey. Categorical variables were summarized by frequencies and percentages, and continuous variables were summarized by means and standard deviations (SD). To compare the mean number of hospital beds and outpatient visits over time between T0, T1, and T2, Friedman's ANOVA was employed with pair comparisons. McNemar’s test was used to identify any change in the organizational measures adopted by AIEOP centers to prevent the spread of the virus between T1 and T2. Wilcoxon test was performed to compare the number of positive cases between T1 and T2. Statistical analyses were performed using SPSS 22.00 (IBM Corp, Chicago). A p-value < 0.05 was considered significant.

## Results

Thirty-six out of 48 centers completed both surveys at T1 and T2 (response rate = 75%). Considering geographical location, 19 centers are located in northern Italy (52.8%), eight (22.2%) in central Italy and nine (25.0%) in southern Italy. Twenty-two centers (61.1%) belong to pediatric hospitals, and 25 out of 36 (69.4%) perform hematopoietic stem cell transplant (HSCT). The majority of respondents were nurses (n = 27; 75%), followed by physicians (n = 5; 13.9%) and other health care professionals (n = 4; 11.1%).

During the pandemic, 33.3% (n = 12) of the participating centers reduced the number of onco-hematology inpatient beds. This reduction was significant over time (p < 0.001), for both T1 and T2 compared to the pre-pandemic period (Table 1), and it was greater for T2 than T1 (p = 0.005). Considering the 24 centers performing HSCT, three (12.5%) reduced the number of inpatient beds dedicated to transplantation. The reduction was significant only between the pre-pandemic period and the second wave of the pandemic (p = 0.026). Moreover, consultations in the outpatient clinics were significantly reduced over time (p < 0.001), for both T1 and T2 compared to the pre-pandemic period, and this reduction was greater for T2 than T1 (p = 0.005).

**Table 1:** Comparison between number of beds/DH before COVID-19, in the first, and in the second wave

Center	Before the pandemic (T0)		First wave of the pandemic (T1)		Second wave of the pandemic (T2)		p*
	M (DS)	Median	M (DS)	Median	M (DS)	Median	
Number of beds inpatient (n = 34)	13.91^a^ (13.99)	10.50	12.38^b^ (14.01)	9.00	11.35^c^ (14.14)	8.50	< 0.001
Number of DH/Ambulatory (n = 32)	58.28^a^ (48.53)	38.50	37.16^b^ (40.21)	25.00	31.72^c^ (38.32)	17.00	< 0.001
Number of beds HSCT (n = 24)	5.13^a^ (2.61)	5.50	4.88^ab^ (2.61) 4.00		4.67^b^ (2.67)	4.00	0.008

*DH* day hospital, *HSCT* hematopoietic stem-cell transplantation

*Friedman's ANOVA; means by line with different superscript letters are significantly different from each other at pair comparisons

Forty-seven percent of the respondents reported that patients did not attend scheduled consultations at outpatient clinics by their own choosing; the main reasons included: consultation considered not urgent (20%), lack of transportation to get to the center because of the reduction in public transport services due to the lockdown rules (15%) and patients presenting flu symptoms (5%). Table 2 and 3 report the organizational strategies, the screening and swab policies adopted by the AIEOP centers to prevent the virus spread among patients and visitors. The specific containment measures to prevent the COVID-19 spread are described in Table 4. The results were compared between T1 and T2.

**Table 2:** Organizational measures adopted by AIEOP centers to prevent the virus spread among patients (n = 36)

	First wave of the pandemic		Second wave of the pandemic		p*
	n	%	n	%	
*COVID-19 pediatric unit*					0.238
Yes	15	41.7	21	58.3	
No	21	58.3	15	41.7	
*COVID-19 unit for pediatric onco-hematological patients*					0.250
Yes	0	0	3	8.3	
No	36	100	33	91.7	
*Area dedicated to the pre-evaluation of pediatric patients in the emergency department*					0.125
Yes	28	77.8	32	88.9	
No	8	22.2	4	11.1	
*Protocols and procedures to manage COVID-19 suspected or confirmed cases*					0.500
Yes	34	94.4	36	100	
No	2	5.6	0	0	
*Protocols and procedures to manage COVID-19 suspected or confirmed cases among the pediatric onco-hematological patients*					0.008
Yes	28	77.8	36	100	
No	8	22.2	0	0	

*AIEOP* Italian Association of Pediatric Hematology and Oncology, *COVID-19* Coronavirus disease 2019

*McNemar’s test

**Table 3:** Screening and swab measures adopted by AIEOP centres to prevent the virus spread among patients and visitors (n = 36)

	First wave of the pandemic		Second wave of the pandemic		p^*^
	n	%	n	%	
*Interview screening before visits/admission*					
Yes, in-person interview (once at the health facility)	6	16.7	5	13.9	1
Yes, phone interview	6	16.7	1	2.8	0.125
Both the above	23	63.9	29	80.6	0.210
No screening performed	1	2.8	1	2.8	1
*Swabs performed before visits in DH*					
Yes, to all the patients	8	8	22	61.1	0.003
Yes, following specific criteria^a^	21	58.3	11	30.6	0.031
No	8	8	3	8.3	0.125
*Swabs performed before admission in the ward*					
Yes, to all the patients	19	52.8	31	86.1	0.004
Yes, following specific criteria^a^	14	38.9	5	13.9	0.049
No	3	8.3	0	0	0.250

*AIEOP* Italian Association of Paediatric Haematology and Oncology, *COVID-19* Coronavirus disease 2019

^a^Criteria included whether patients were symptomatic (fever and cough) or coming from the “red zones”

*McNemar’s test

**Table 4:** COVID-19 containment measures adopted by AIEOP centres to prevent the virus spread among patients and visitors

	First wave		Second wave		p*
	n	%	n	%	
Limit of one visitor/one caregiver per patient	34	94.4	24	66.7	0.013
Recreational activities by volunteers, clown therapists and play therapists suspended	32	88.9	21	58.3	0.003
Forbidden visits from external people	31	86.1	21	58.3	0.013
Signage dedicated to COVID-19 spread containment throughout the facilities	31	86.1	27	75.0	0.388
Redesigning of facilities, common areas, and pathways	31	86.1	23	63.9	0.077
More frequent sanitation of the areas dedicated to healthcare activities	30	83.3	19	52.8	0.035
Wearing mask as an essential criterion to enter the facilities	29	80.6	28	77.8	1
Alcohol-based hand rub available in the common areas	29	80.6	29	80.6	1
Institution of digital in-hospital schooling	24	66.7	21	58.3	0.629
Making masks available to those who enter the facilities	22	61.1	24	66.7	0.791
Hospital cafeteria closed to the public (visitors)	21	58.3	10	27.8	0.007
Body temperature measured on those who enter the facilities	19	52.8	22	61.1	0.629
Phone number dedicated to answer COVID-19 related questions from families and patients	17	47.2	5	13.9	0.002
Online and digital resources available for patients’ playtime and recreational activities	15	41.7	10	27.8	0.180
Shops in hospital closed to the public (visitors)	12	33.3	7	19.4	0.227
Use of thermal scanner for those who enter the facilities	10	27.8	19	52.8	0.049
Hospital canteen closed to the public (visitors)	11	30.6	9	25.0	0.754
Available gloves to those who enter the facilities	11	30.6	9	25.0	0.774
Wearing gloves as an essential criterion to enter the facilities	9	25.0	1	2.8	0.021

Multi-response items

*COVID-19* Coronavirus disease 2019, *AIEOP* Italian Association of Paediatric Haematology and Oncology

*McNemar’s test

### COVID-19 Confirmed Cases Within the AIEOP Network

From data collected through the two surveys during the second wave of the COVID-19 pandemic, a total of 174 pediatric patients with cancer were tested positive for SARS-CoV-2 versus 37 patients during the first wave. This five-fold increase in the number of positive patients was significant (p < 0.001) (Table 5). Half presented mild symptoms and were managed at home. Forty percent were admitted to infectious disease or COVID-19 dedicated units, and 13% to the AIEOP onco-hematology unit. One patient was admitted to Intensive Care Unit (ICU). Fifteen percent of these patients underwent chemotherapy or HSCT. Similarly, the number of COVID-19 positive physicians increased fivefold between the first and the second wave of the pandemic, from a total of 19–91 cases (p < 0.001), and the number of positive nurses increased fourfold from a total of 36–150 (p = 0.005) (Table 5). However, the number of assistant nurses being tested positive did not change significantly between the two waves, with a small increase from 11 to 26.

**Table 5:** Comparison of the number of COVID-19 positive cases between the first and the second wave of the COVID-19 pandemic

Centre	First wave of the pandemic		Second wave of the pandemic		p*
	M (DS)	Median	M (DS)	Median	
Positive inpatients	1.03 (2.04)	0.00	4.97 (7.48)	2.00	< 0.001
Positive physicians	0.53 (1.06)	0.00	2.53 (4.05)	1.00	< 0.001
Positive nurses	1.00 (1.74)	0.00	4.17 (7.31)	2.00	0.005
Positive assistants	0.31 (0.75)	0.00	0.72 (1.09)	0.00	0.66

*Wilcoxon test

## Discussion

The study described and compared at a national level the measures implemented in 36 Italian pediatric onco-hematology units within the AIEOP network and the number of infections among patients and healthcare staff during the first and second wave of the COVID-19 pandemic.

According to the official registry, the participating centers proportionally cover almost all of the annual pediatric onco-hematological diagnoses in Italy, as the 88% of the annual diagnoses take place in these centers [9]. The data confirmed a significant reduction in the number of hospital beds, especially between the pre-pandemic period and the second wave of the pandemic. This reduction could be attributed to a reorganization of hospital beds in order to deal with the emergency situation. Given the excessive number of COVID-positive patients, some units were completely reorganized by converting from onco-hematology to COVID Units. In addition, the number of HSCT beds was significantly reduced in the first wave of the pandemic but remained unvaried in the second wave. Border closures and international movement restrictions led to a slowdown in the activity of HSCT in Italy. The main issue was related to the import of stem cells, especially from abroad, since the entire continent was in lockdown [10]. This situation resulted in a significant reduction in transplant activity during the pandemic [11].

Significant reductions were also recorded in the number of outpatient clinic visits between the pre-pandemic period, the first wave, and the second wave of the pandemic. This was done to limit the exposure of fragile pediatric patients to the SARS-CoV-2 virus, and was also a consequence of families becoming fearful to attend hospitals, in line with what happened to other patients affected by other pathologies [10, 12–15]. During the second phase of the pandemic, this reduction could also be attributed to the implementation of alternative methods of medical assistance, such as telemedicine and remote telemonitoring [16]. It is important to ensure continuity of care for pediatric patients with cancer, as studies on adult patients have shown that the reduction of hospital services led to a delay in cancer diagnosis, causing malignancies to be detected only at an advanced stage, with a higher risk of complications [17].

Many organizational strategies were put in place during the first and second waves of the pandemic to avoid the spread of the virus. In particular, COVID-19 units exclusively dedicated to pediatric patients were implemented in the first wave and were maintained in the second, while three centers developed a dedicated COVID-19 pediatric onco-hematology unit in the second wave. Most of the AIEOP centers created dedicated pathways for the pre-evaluation of suspected or COVID positive pediatric patients accessing the emergency departments. The situation improved after the first wave, in which there were tents installed in emergency departments and well-defined and structured pathways within the hospitals. Furthermore, the number of centers which defined specific protocols and procedures for the management of suspected and/or confirmed COVID-19 cases for onco-hematology pediatric patients significantly increased between the first and second waves of the pandemic. The AIEOP centers that initiated these procedures, that were established during the second wave of the pandemic, focused on attentive care to these specific patients [5, 18–20]. This also shows the great adherence of the centers to the recommendations of the AIEOP scientific society on March 20, 2020 [6].

Our findings show how AIEOP centers implemented phone and/or on-site screening for patients and their carers both in the first and second wave of the pandemic. Screening is an extremely effective tool, and therefore highly recommended [21]. Another important measure implemented in the AIEOP centers was the nasopharyngeal swab for patients. In particular, the number of AIEOP centers performing swabs on patients in the outpatient and inpatient facilities increased between the first and the second wave of the pandemic. This may suggest a more pro-active approach in the fight against the SARS-CoV-2 virus.

The AIEOP centers implemented the international recommendations regarding the use of standard precautions [22], the use of face masks, hand washing with hydroalcoholic gel, social distancing and isolation, both in the first and second wave of the pandemic. In particular, the thermal scanner was further implemented in the second wave compared with the first, while the use of gloves as an essential criterion to enter the facilities decreased. Similarly, the number of centers limiting the allowance for only one parent/carer to enter for each child was diminished [5]. This may underline the unnecessary use of this restrictive measure, once the correct precautions are in place [23]. On the other hand, the number of AIEOP centers prohibiting access to volunteers, educators, teachers, and external people decreased between the first and second wave of the pandemic. This may be due to the introduction of new leisure and training activities to help children to better live hospital isolation [24]. Indeed, many projects have been implemented in AIEOP centers: in-hospital schooling went digital, web platforms were created to host recreational meetings led by volunteers and clown-therapists. Virtual meetings with psychologists and play-therapists have been promoted by some of the respondents. These new strategies would deserve further investigation on their efficacy and future feasibility [4].

We observed a five-fold increase in pediatric patients who tested positive for COVID-19 between the first and the second waves of the pandemic. It is important to note that their general clinical conditions were good with only mild symptoms. This increase can partially be attributed to Italy’s more relaxed lockdown measures for the second wave of the pandemic compared to the first, which allowed a greater spread of the virus over the entire population, as more contagious variants of the virus were spreading [3, 25]. Although international studies report that children with cancer show a high tolerance to the infection without the presentation of dangerous complications [17, 26, 27], health centers should still be vigilant in preventing the infection in this population. For example, patients and caregivers should be educated on a healthy lifestyle that involves the adoption of basic infectious and protective control practices [28, 29].

In 85% of cases the infection was probably contracted outside the workplace, the five-fold increase in the number of cases between the first and second waves of the pandemic is concerning, and may reflect the vulnerability to the SARS-CoV-2 among health professionals [30].

## Limitations

This study’s findings should be read in light of certain limitations. First, data were collected using an online survey. Second, the findings reflect an Italian context, with a survey response rate of 75%, and need to be confirmed among other countries. Third, data about positive cases among health professionals were not compared with epidemiological data. Moreover, our study was merely descriptive: to date, we have not empirically tested which measures were most effective. Finally, we could not relate the measures implemented with the patients’ outcomes, including delayed diagnoses [31, 32].

## Conclusions

The new coronavirus is representing a health, social, economic, and even ethical challenge. All AIEOP centers showed the capacity to adapt and promptly respond to the exceptional pandemic situation. In particular, between the first and the second waves of the pandemic, the AIEOP centers maintained most of their preventive measures implemented during the emergency phase. Therefore, they managed to avoid the catastrophic consequences of this pandemic, preventing the worst from happening. Indeed, the AIEOP centers were already familiar to adopting preventive measures for infectious risk, since they routinely treat patients potentially at risk (i.e., immunocompromised, neutropenic, post-transplant) [33]. These measures, partly in line with the recommendations to limit the spread of the coronavirus, could have helped the centers limit the number of infections. However, SARS-CoV-2 has spread among patients and health professionals, therefore we need to remain alert and promote vaccinations. The current health crisis has led to global preparations for future pandemics or emergencies, limiting effects and further consequences as much as possible. In particular, based on our experience and in line with AIEOP recommendations [6], the most important measures to be taken into account in similar situations include the immediate activation of hospital protocols dictating screening measures, pathways dedicated to pediatric onco-hematology patients within the hospital, home care services and remote assistance via telemedicine to ensure continuity of care and access to healthcare services. In addition, it is crucial to invest in staff training, to ensure a minimum supply of PPE and to provide targeted education to patients and caregivers about hygiene and protective isolation measures.
